# On the Top-Down Argument for the Ability to Do Otherwise

**DOI:** 10.1007/s10670-022-00638-3

**Published:** 2022-11-22

**Authors:** Leonhard Menges

**Affiliations:** Department of Philosophy (GW), Salzburg University, Kapitelgasse 4-6, 5020 Salzburg, Austria

## Abstract

The Top-Down Argument for the ability to do otherwise aims at establishing that humans can do otherwise in the sense that is relevant for debates about free will. It consists of two premises: first, we always need to answer the question of whether some phenomenon (such as the ability to do otherwise) exists by consulting our best scientific theories of the domain at issue. Second, our best scientific theories of human action presuppose that humans can do otherwise. This paper argues that this is not enough to establish the conclusion. The Top-Down Argument supports that humans can do otherwise *in some sense*. But it does not show that humans can do otherwise *in the sense that is relevant for debates about free will*. The paper then shows that the apparently best way to make the argument valid does not work.

## Introduction

Many think that having free will requires the ability to do otherwise. Let us assume, for the sake of the argument, that this is so: if people can’t do otherwise–in a sense to be explored below––then they lack free will. Some think that humans cannot do otherwise in our world and, therefore, do not have free will. They may argue that physical determinism or the fact that all human actions and decisions are a matter of luck beyond human control or God’s foreknowledge undermine people’s ability to do otherwise.

Recently, List (2014, sec. 5; 2019, 97–103) has developed the Top-Down Argument to the conclusion that humans, in our world, can do otherwise and, thereby, fulfill a contested necessary condition for having free will. A key component of this argument is that the human ability to do otherwise is supported by scientific inquiries into human action. This is especially interesting because a common theme in public discourse is that scientific explanations of human conduct pose a threat to the idea that humans can do otherwise and have free will (e.g., Harris, 2012).

The Top-Down Argument starts from an assumption that is well known from debates about scientific realism and that List calls the naturalistic ontological attitude (List, 2019, 74). The core idea is that when we ask whether some entities or phenomena exist, we should look at our best scientific theories of the relevant domain. If these theories imply that the entities do exist, then we should take this implication at face value and conclude that they are real. For example, if our best physical theories imply that the Higgs boson and gravitational forces exist, then asking “whether they are ‘really’ real would be to ask one question too many” (List, 2019, 74).

List applies this line of thinking to free will. Here, we are concerned with human action and behavior. For List, the best scientific theories of this domain come from cognitive and economic psychology. These theories aim at predicting and explaining why agents act the way they do. The explanations work by saying what it is about agents that explains why they opt for one option rather than another where both options are available to them; for example, why people go vote rather than stay home. The best answers refer to the intentional states of the agents, such as their beliefs and desires. These intentional states are taken to explain why one option is realized and not the other, even though the other could have been realized (see List, 2019, 62, 99). Thus, according to List, our best scientific theories of the domain of human conduct imply that humans’ intentional states explain why they perform one action instead of a different one. These theories presuppose that humans have intentional agency, which enables them to choose between different options, that is, that humans can do otherwise.

Here is a summary of the Top-Down Argument:

1. “Naturalistic ontological attitude: Our best guide to any questions about which entities, properties, or phenomena exist in any given domain is to be found in our best scientific theories of that domain” (List, 2019, 74).
2. “Indeterminism in psychology: Our best theories of human agency, in areas ranging from cognitive psychology to behavioural economics, presuppose that people face choices between more than one course of action. For these theories, an important goal is to explain how people make such choices, not to explain the phenomenon of choice making away” (List, 2019, 98).
3. Therefore, the best answer to the question of whether people (at least sometimes) face choices between more than one course of action is “Yes”.
4. Therefore, we have sufficient reason to believe that people (at least sometimes) can do otherwise.
5. Therefore, we have sufficient reason to believe that people (at least sometimes) fulfill one contested necessary condition for having free will.

I will argue that this argument relies on a substantial hidden premise (Sections 2 and 3) and that there are good reasons to reject it (Sect. 4 ). I will not discuss List’s argument to the conclusion that the ability to do otherwise, as it is defended by the Top-Down Argument, is compatible with physical determinism. This line of thinking has already generated an interesting debate (see Elzein & Pernu, 2017; Gebharter, 2020; Menges, 2021). I will also not discuss the question of whether this account of free will has a problem with luck (see Mele, 2020). The worry I will raise is more fundamental. It asks: is the ability to do otherwise that is defended by the Top-Down Argument relevant for the free will debate?

## Free Will: What’s at Issue?

In this section, I will first argue that the debate about free will needs a common understanding of free will that is, as far as possible, non-committal with regard to substantial questions about, for example, compatibilism or incomaptibilism or about what, exactly, free will consists in. Then, I will present such a non-substantial understanding that is widely but not universally accepted and that characterizes free will in terms of praise- and blameworthiness. In the next section, I will show how this is relevant for the Top-Down Argument.

In everyday thinking and talking, there are very different contexts in which it makes sense to say of agents that they do or do not face choices, can or cannot do otherwise. Victims of a robbery may not face a choice in some sense of “facing a choice” when a gun is pointed at their head. Luther said of himself “Here I stand, I can do no other” when he was ordered to take back his criticism of the Catholic Church. This may have been true in some sense of “I can do no other”. When people steal your money because they have kleptomania, then they seem to lack the ability to do otherwise in some sense of “ability to do otherwise”.

Not all of these senses of “facing choices” and “can do otherwise” are relevant for free will. We understand what Luther means when he says “I can do no other” without having to assume that, if he is right, he has no free will. Evidence for this is that we may take Luther to be responsible and even praiseworthy for not taking back his criticism, which, according to many theories, including List’s (see List, 2019, 28), presupposes that he has free will. Luther seems to be saying that the topic at issue is so important to him that he can hardly imagine himself exercising his will by renouncing his criticism (see List, 2019, 24). Similarly, we can understand what victims of a robbery mean when they say that they could not have acted otherwise than hand over the money, without having to ascribe the thought to them that they had no free will. Again, we may think that they are responsible and praiseworthy for coolly handling this difficult situation, which suggests that they did it out of free will. Instead of denying free will, the victims seem to be saying that they had no reasonable alternative or fairly curtailed options, which partly explains and justifies their conduct.

If this is on the right track, then there are senses of “can do otherwise” that are irrelevant for free will: you may not be able to do otherwise in these senses and still have free will, such that these senses of “can do otherwise” have nothing to do with the contested agency condition for free will.^1^ Correspondingly, defenders of the idea that humans in our world can do otherwise in the way that is relevant for free will are not successful just because they have shown that, in our world, humans have *some sort* of ability to do otherwise. This is so because the ability they have defended may not be the one that is relevant for free will (I will come back to this idea in the end of Sect. 3).

This brief discussion is meant to show that the debate about free will needs a common understanding of what should be at issue. That is, we need a characterization of free will and the ability to do otherwise that is, as far as possible, neutral with regard to contested substantial questions about, for example, compatibilism or incompatibilism or about what, exactly, free will consists in. Theorists who disagree on these and other questions should be able to accept what I will call a *non-substantial characterization of free will*. Then, they can be sure that they are talking about the same thing when they discuss, for example, if free will and the ability to do otherwise, thus understood, is compatible with determinism (thanks to an anonymous referee for pressing me on how this characterization should be understood).

Although there is no universal agreement on a non-substantial characterization of free will, many authors characterize the sense of free will they are concerned with in terms of moral responsibility and they characterize moral responsibility in terms of praise- and blameworthiness (for an overview, see the recent debate between McKenna, 2019a, 2019b; Pereboom, 2019; Nelkin, 2019). To illustrate, take the case of the person who steals your money because of an impulse control disorder. It seems that this person lacked an important kind of agency or control in some sense of these terms that is relevant for the question of whether the person is blameworthy for stealing money. It would be natural to think that blaming the person would be in some everyday sense undeserved.

Perhaps (I do not take a stance on this) there is a sense of desert, according to which it would be deserved to blame the person if blaming the person has better consequences than not blaming or if the person has consented to being blamed in advance. However, there clearly is an everyday notion of desert and a corresponding notion of blame- and praiseworthiness that work differently. According to these notions, your colleagues deserve praise just because they freely and knowingly helped you organize a conference, and not because praising them has good effects. Similarly, these are the notions we use when we believe that our neighbors deserve blame just because they freely partied all night long even though they knew that it would keep us and our children awake and ruin the next day. They deserve blame, the everyday idea goes, just because of what they did and how they were, not because blaming them has good consequences or because they agreed to being blamed. In the same sense, the person with an impulse control disorder seems not to deserve blame for stealing even if blaming the person has good effects, such as deterring others from stealing in the future.

In the philosophical debate, the everyday notions I have just relied on are often called blame- or praiseworthiness in the basic-desert sense (see, again, the debate between McKenna, Pereboom, and Nelkin in *The Journal of Ethics*).^2^ The expression “basic desert” is often associated with Pereboom’s (e.g., 2014) skeptical theory of free will and responsibility. However, the expression is meant to be non-committal regarding questions about skepticism, compatibilism, or the nature of the agency that constitutes free will. That is, “basic desert” is meant to capture the notion at issue in the everyday thoughts that our neighbors deserve blame, our colleagues deserve praise, and the person with the impulse control disorder does not deserve blame in the cases sketched above. These everyday thoughts are independent of any specific theory of how to understand the agency that these people need to have or lack for the thoughts to be true. However, they imply that *some* agency is necessary for blame- and praiseworthiness. Moreover, they imply that this agency alone is not sufficient for blame- or praiseworthiness. However, when this agency is combined with some other agential features, then they together are sufficient for praise- or blameworthiness. More specifically, and to focus on blameworthiness, when an agent is blameworthy for an action in the basic desert sense, then this is because the action is problematic (e.g., morally impermissible), the agent fulfills some epistemic condition (e.g., has sufficient reason to believe that the action is impermissible) and some agency condition. Fulfilling these conditions is necessary and sufficient for basically deserved blame. We are committed to this when we think that our neighbors deserve our blame just because of what they did and how they were when they did it and independent of any considerations about consequences.

Let me make two notes in order to avoid misunderstandings. First, claims about blameworthiness in the basic desert sense do not imply that blame is all-things-considered acceptable. For example, it may be that our neighbors basically deserve blame for keeping us awake all night while it would be all-things-considered impermissible to blame them, perhaps because this would have catastrophic consequences for our family and other neighbors. Second, holding that someone is blameworthy in the basic desert sense does not necessarily imply the retributive idea that there is something non-instrumentally good about harming this person (an idea that List explicitly rejects, see his contribution to List et al., 2020). The idea of basic desert can be spelled out in ways that avoid reference to the value of harm or punishment (see, e.g., Levy, 2011, 3; Nelkin, 2016, sec. 2). In what follows, I always mean this basic desert sense when I talk about praise- or blameworthiness.

Many authors use the notions of blame- and praiseworthiness in the basic desert sense in order to characterize the kind of free will they are concerned with. They accept as an as far as possible non-substantial background assumption that free will should be thought of as the strongest kind of agency that is relevant for blame- and praiseworthiness in the basic desert sense. This characterization is non-substantial in the sense that it does not preclude any of the major views on what free will consists in––say, sourcehood or the ability to do otherwise––and on whether it is compatible with determinism. It is, nonetheless, helpful because it provides the ground on which these substantial questions can be fruitfully discussed without talking past each other. To see this, note that agnostics about compatibilism such as Alfred Mele (2019, chap. Introduction), libertarians such as Christopher Franklin (2018, chap. 2), compatibilists such as Dana Nelkin (2011, chap. Introduction), Carolina Sartorio (2016, chap. 1), and David O. Brink (2021, chap. 1), and revisionists such as Manuel Vargas (2013) work with the characterization of free will as the strongest agency that is relevant for basically deserved praise or blame (note, however, that Vargas’ justification of desert has a forward looking element that may come in conflict with how other authors understand the notion; see, e.g., Vargas, 2015, sec. 2). Perhaps most importantly for our context, this is the sense of free will those have in mind who are skeptical about free will (see, e.g., Strawson, 1994; Levy, 2011; Waller, 2011; Pereboom, 2014; Caruso & Morris, 2017; for an overview, see Caruso, 2018).

To briefly sum up, in order to make sure that authors in the free will debate are talking about the same thing, we need a common, non-substantial characterization of free will. Many authors adopt an understanding, according to which free will can be characterized as the agency condition that is relevant for a specific kind of moral responsibility.

## The Missing Premise

With this characterization of free will in mind, let us go back to List’s view. According to him, having free will involves, first, having intentional agency that, second, causally explains one's actions and, third, having the ability to do otherwise (see List, 2019, 22–27). Moreover, he agrees with the idea that free will is “a necessary condition for a salient form of moral responsibility” (List, 2019, 28).

If one combines this picture with the non-substantial characterization of free will presented in the preceding section, then the ability to do otherwise has a very specific role to play: its presence explains why an agent is blameworthy when all other relevant moral, epistemic, and agency conditions are fulfilled and its absence guarantees that the agent is not blameworthy. To illustrate, imagine that someone impermissibly steals your money (moral condition), that the agent had sufficient reason to believe that this is impermissible (epistemic condition), and that the agent's intentional agency causally explains the act of stealing your money (part of the agency condition). Then we know, according to the picture sketched above, that some important conditions for blameworthiness are fulfilled. The question of whether the person basically deserves blame is still not settled, though. It may be that the agent does not have the ability to do otherwise in a sense that is relevant for blameworthiness. If the agent has the relevant ability, then the person basically deserves blame. If the agent lacks the ability, then the person does not basically deserve blame. Let me call the ability to do otherwise that plays these roles the *strongest ability to do otherwise relevant for praise- and blameworthiness in the basic desert sense*.

Note that the strongest ability to do otherwise relevant for blame- and praiseworthiness is only characterized in terms of the roles it plays in everyday moral thinking: its absence explains that an agent is neither blame- nor praiseworthiness; its presence explains, when combined with other features, blame- or praiseworthiness. This characterization is neutral with regard to questions about skepticism, compatibilism, and the exact nature of the ability to do otherwise. Authors who give very different answers to these questions are in the position to accept the characterization.

Now, let us look at the Top-Down Argument again. Premise (2) says that the best scientific approaches to human action presuppose that people face choices. Let us grant this. But we have seen that we can sensibly say of people in very different contexts that they do or do not face choices and that discussions about free will skepticism are typically concerned with a specific context. They focus on the strongest kind of agency that is relevant for blame- or praiseworthiness in the basic desert sense. Thus, it would be too quick to directly infer from the idea that people can do otherwise, as premises (1) and (2) suggest, to the conclusion that people fulfill a contested necessary condition for free will, as (C3) says. In order to make the argument relevant for skeptical challenges, one needs an additional premise. It would say that the sense of “can do otherwise” and “facing choices” that is at issue in premise (2) is the one that is at issue in debates about free will skepticism. More precisely, the Top-Down Argument relies on the following Missing Premise:

(MP) The ability to do otherwise and the kind of facing choices that is presupposed by the best scientific explanations of human action is the strongest ability to do otherwise relevant for praise- and blameworthiness in the basic desert sense.

Let me elaborate. MP relies on the idea that the social and psychological sciences presuppose that humans can do otherwise, as premise (2) of the Top-Down Argument says. The Missing Premise says that the sense of “can do otherwise” that is presupposed by these sciences is the sense of “can do otherwise” that is relevant for basically deserved praise or blame. That is, its absence guarantees that the agent is neither blame- nor praiseworthy. But if the action is impermissible, the agent had sufficient reason to believe this, the agent's intentional agency causally explains the performance of the action, *and if the agent has the ability to do otherwise that is presupposed by the best scientific explanations of human action*, then the agent is blameworthy for the action––or so the Missing Premise says.^3^

To avoid misunderstandings, the point of the discussion so far is *not* that proponents of the Top-Down Argument are logically committed to the Missing Premise. They aren’t. As I said in Sect. 2, not everybody in the free will debate accepts the characterization of free will or the ability to do otherwise in terms of blame- or praiseworthiness. Some suggest or explicitly say that they are not (primarily) concerned with moral responsibility and the basic-desert justification of praising or blaming but with something else (van Inwagen, 1983, sec. 1.4; Kane, 1996, chap. Introduction; Campbell, 1997; Berofsky, 2012, chap. 1; Vihvelin, 2013, chap. 1). Proponents of the Top-Down Argument can side with them and deny MP. However, if proponents of the Top-Down Argument refuse to add MP, then they are talking past an important number of influential free will theorists, some of whom I have already cited above, including Mele, Nelkin, Pereboom, Sartorio and others. It would be unclear how proponents of the Top-Down Argument relate to what these theorists say about free will and can do otherwise.

One may try to make the relation between these authors and proponents of the Top-Down Argument who deny the Missing Premise clearer by proposing the following: in contrast to the authors I have just listed, proponents of the Top-Down Argument only aim at showing that humans in our world can do otherwise in *some* sense of “can do otherwise”. Therefore, the proposal says, they can leave it open whether this sense is the one that is relevant for blame- and praiseworthiness (thanks to an anonymous referee for this suggestion).

However, this reply is unavailable to the proponent of the Top-Down Argument. We have seen in the beginning of the preceding section that there are senses of “can do otherwise” that are irrelevant for free will, such as Luther’s ability or inability to renounce his criticism of the Catholic Church. Proponents of the Top-Down Argument would not have succeeded if they could only show that humans have the ability to do otherwise that Luther is supposed to lack. By their own lights, they need to defend the idea that humans can do otherwise in a *specific* sense of “can do otherwise” that is clearly relevant for free will.^4^ If they deny MP and, thereby, the idea that they are concerned with the same sense of “can do otherwise” that is at issue in the theories of Mele, Pereboom, Sartorio, and so on, then it is unclear how the Top-Down Argument relates to these authors and their arguments.

Even more problematically, proponents of the Top-Down Argument would talk past the current skeptics and their challenges if they denied the Missing Premise. Free will skeptics are skeptical about a very specific aspect of agency that one can identify by describing its role in an everyday justification of praising and blaming people (for an overview, see Caruso, 2018). If proponents of the Top-Down Argument for the ability to do otherwise are concerned with some other aspects of agency, then there is no dispute between them and the skeptics. For those who are concerned with and perhaps even worried because of current skeptical arguments, the Top-Down Argument would lose much of its initial attractiveness. It would be of no help to meet the skeptical challenges. Thus, if the Top-Down Argument is taken to engage with the most important current free will skeptics, then one needs to add the Missing Premise.

To sum up, the original Top-Down Argument is incomplete if one assumes that it aims at engaging with the most powerful skeptics about free will and many important non-skeptical free will theorists. In order to make the argument relevant for the current discussions about skepticism, one needs to add the Missing Premise. In the remainder of the paper I will argue that there are good reasons to be skeptical about the Missing Premise.

## The Implausibility of the Missing Premise

For making sure that the Top-Down Argument engages with skeptical challenges, one needs to show that scientific explanations of human conduct presuppose that humans have a very specific ability to do otherwise: the ability that makes an agent blameworthy who fulfills the other conditions identified above. In other words, proponents of the argument should show that the scientific approach to explaining human conduct is concerned with exactly the same kind of ability to do otherwise and having choices that we are concerned with when we ask whether people deserve to be blamed or praised just because of what they did and how they were when they did it.

A first worry about this claim is that it would be a glaring coincidence if both approaches really are concerned with the same ability. This is because, as we have seen above, in different contexts different abilities to do otherwise are relevant. For example, the ability to do otherwise that we lose when robbers point their gun at our heads is not the strongest ability that is relevant for blame- or praiseworthiness. The victims can be praiseworthy for coolly handling the situation. The Missing Premise implies that our adopting the purely empirical, non-normative perspective that aims at explaining and predicting human action requires us to assume that humans have a certain ability *X*; and our adopting the desert based perspective that aims at evaluating humans in terms of blame- or praiseworthiness requires us to ask whether humans have exactly the same ability *X*. And this is so even though we know that importantly different abilities go by the same name “*X*”. This suggests that accepting the Missing Premise comes with a relatively heavy burden of justification. As long as there is no good positive argument for it, we have good reason to be skeptical about it.

In addition to this burden-shifting consideration, there is more direct reason to reject the Missing Premise. The scientific explanation of human action requires a less demanding ability to do otherwise than the strongest ability to do otherwise that is relevant for praise- and blameworthiness. Thus, people may have the ability to do otherwise that makes them a proper object of scientific inquiry, without having the strongest ability to do otherwise that is relevant for blameworthiness. Let me elaborate.

Imagine that 9% of the electorate in your community voted for an extremist right-wing party instead of not voting or voting for other parties. You are having a nice dinner with your liberal friends from the same community and one of them angrily blames those who voted for the extremist right-wing party. The question comes up: are they blameworthy? Note that we should distinguish this question about blameworthiness from whether your friend has the standing to blame or whether it is all-things-considered permissible to blame them. The answer to the latter two questions may very well be “No”, while the answer to the former may be “Yes”. Here, we are only concerned with whether the voters fulfill the conditions for basically deserved blame. Let us imagine that the following is true and we know it to be true: first, voting for the right-wing party is impermissible. Second, the newspapers, radio shows, online blogs, and so on in your community provided good evidence for the conclusion that this is so and the epistemic abilities of the voters are largely intact such that they had sufficient reason to believe that voting for the party is impermissible. Third, their voting for this party is causally explained by their intentional agency. Thus, let us imagine that we know that the voters fulfill most of the conditions of blameworthiness. However, we still wonder whether they fulfill the ability-to-do-otherwise condition that is relevant for blameworthiness.

Now imagine that another friend of yours is a social scientist who has studied the voting behavior in your community by adopting the best methods of political science, behavioral economics, and cognitive psychology, including analyzing exit polls, running experiments with a representative group of voters, conducting in-depth interviews with them, and so on. Imagine that the voters were fully cooperative. Your friend came up with a mix of partial explanations that involve people’s dissatisfaction with more traditional parties, their right-wing beliefs and commitments, fears of social change, and so on. This scientific explanation of human conduct presupposes that the voters faced choices. It aims at explaining why they voted for one party rather than one of the others. It does not try to explain the phenomenon of choice making away (see List, 2019, 98). Thus, we should conclude that the voters could have voted for another party in some sense of “could have voted for another party”.

If the Missing Premise is correct, then the question of whether the voters are blameworthy is settled now: we already knew that they fulfill the epistemic condition and that their intentional agency was causally relevant (and we assume that this is relevant for blameworthiness). Now we know that their behavior is explainable by the best scientific methods. The Missing Premise says that this implies that they fulfill the strongest can-do-otherwise condition that is relevant for blameworthiness. Thus, they are blameworthy.

However, it is too early to draw this conclusion. So far we have learned that certain beliefs, values, and emotions partially caused their voting behavior. But this only rules out some excusing explanations of their behavior that were not very probable from the start, for example, that they were mindless marionettes or forced to vote as they did. However, that they had certain values, beliefs, and emotions that partly caused their voting behavior does not tell us much about whether they could have voted for another party in the sense that is relevant for deserved blame. This is because there may have been factors present that seem to undermine the ability to do otherwise that is relevant for blameworthiness. Perhaps an evil neuroscientist manipulated the brains of some of them and implemented the intentional states that caused their voting for the extremist party. Perhaps some of the voters are victims of decades-long propaganda that caused them to develop the relevant intentional states. Perhaps some of them are victims of an unjust society which explains why they have the values that they in fact have.

Now, one can go into complex discussions about each of the factors and ask whether it would undermine the ability to do otherwise that is relevant for blameworthiness. I will not do it. Instead, imagine for a moment that there is a group of voters for whom the following is true: they belong to a group that were radically discriminated against *and* they are the object of effective political propaganda *and* they are manipulated by evil neuroscientists. Intuitively, this complex background undermines the voters' ability to do otherwise that is relevant for blameworthiness. This intuitive thought, however, is in conflict with the Missing Premise. The presence of all these seemingly blameworthiness-undermining factors is compatible with the truth of the scientific explanation of their voting behavior and their fulfilling the other conditions listed above. Insofar the scientific approach presupposes that the voters faced choices, they did face choices in some sense. The Missing Premise says that this sense of facing choices is the one that is relevant for blameworthiness, and therefore, there is, according to MP, absolutely no reason to have doubts about the voters’ blameworthiness-relevant ability to do otherwise. However, there is reason to have doubts about their having this ability. Therefore, the Missing Premise is implausible.^5^

Proponents of the Top-Down Argument will probably not be convinced (thanks to an anonymous referee for pressing me on this). One thing they could say is that studying the voters I have just presented may also *reveal* that they were manipulated, discriminated against, and the object of propaganda. This is because it is partly an empirical fact about their agency that they were treated this way. The social sciences provide us with the best methods to learn about these facts. Thus, a complete psychological, economical, and political study would not only tell us about their fears, values, and beliefs, it would also tell us about their being manipulated, the object of propaganda, and the victim of discrimination.

This is true, but it does not save the Missing Premise and the Top-Down Argument. To see this, note that we have two different groups of studies here. The first asks, roughly, “which intentional states explain the voting behavior?” The relevant studies answer this question by pointing to beliefs, values, fears, and so on. The second group asks, roughly, “what explains that the voters have the intentional states that explain their voting behavior?” The answers point to discrimination, manipulation, and propaganda. Both explanations are consistent with each other. That is, it may be true that the voters’ beliefs, fears, and values explain their voting behavior and that discrimination, manipulation, and propaganda explain why they have these beliefs, fears, and values. Importantly, the second explanation does not imply that the intentional states of the voters are causally irrelevant or that they play no role in the best explanation of their voting behavior.

According to premise (2) of the Top-Down Argument, the truth of the first explanation implies that the voters had a choice and could have done otherwise. That is, if the first explanation is true and the voting behavior is explained by the intentional states of the voters, then they had a choice between voting for the extremist party and something else (not voting or voting for another party). Thus, if the best methods of the social sciences explain agents’ conduct by referring to their intentional states, then the agents can do otherwise in the sense that it relevant for the Top-Down Argument. This is so independent of why they have these states. Therefore, studies that reveal that the voters were manipulated, discriminated against, and the object of propaganda do not necessarily undermine the claim that they had a choice in the sense that is relevant for the Top-Down Argument. Combining the Top-Down Argument with the Missing Premise thus implausibly implies that these voters are clearly blameworthy.

The general idea that this case is meant to make clear is that the agency that is relevant for basically deserved blame is more demanding than the ability to do otherwise that is presupposed by the best scientific explanation of human action. Humans may be the proper objects of psychological, economic, or other studies of the social sciences without fulfilling the agency conditions that is at issue in current discussions about free will skepticism. The Missing Premise, however, says that scientific explanation and deserved blame require the same ability to do otherwise. Therefore, we have good reason to reject the Missing Premise.

The deep question now is what exactly blame- and praiseworthiness in the basic desert sense presuppose. The short answer is: I do not know. This paper is not the place to even try to answer the deep question. However, the list of potentially undermining factors suggests where one could look for an answer. Perhaps the kind of agency that is necessary for blameworthiness requires, among other things, that the intentional states of the agent have a certain history and are not the product of certain freedom-undermining factors (this is what historicism or externalism about free will and responsibility says; for an overview see McKenna and Coates 2021). Again, this is not the place to go into details. What is important for the aims of this paper is that the core idea of the Top-Down Argument will not do the job: it is not enough to look at what scientific explanations of human action presupposes to identify the kind of agency that is relevant for free will.

## Conclusion

The Top-Down Argument tries to infer that humans can do otherwise in the sense that is relevant for free will from the claim that the scientific explanation of human action presupposes that humans face choices and from the idea that questions about what there is should be answered by our best scientific theories. This argument is especially interesting because it turns around the typical line of thinking that a purely scientific perspective is in conflict with believing in free will. However, I have argued that the argument is not successful. If the Top-Down Argument aims at engaging with the most powerful opponents of the idea that humans have free will, then it must be complemented by the additional premise that the ability to do otherwise that is presupposed by the scientific explanation of human conduct is the same ability that is relevant for blame- and praiseworthiness in the basic desert sense. However, there is no argument for this premise and there is good reason to have doubts about it. If proponents of the Top-Down Argument refuse to add this premise, however, then they will talk past the strongest opponents of free will and many other free will theorists. Both options look bad.^6^

## Footnotes

1 Interestingly, List suggests that so-called dispositional and conditional interpretations of “can do otherwise” also identify senses of “can do otherwise” that are irrelevant for free will (List 2019, 85).

2 McKenna (2019b) sketches a way to characterize free will in terms of moral responsibility without characterizing responsibility in terms of basic desert. I cannot go into the details here, but it seems to me that the problems for the Top-Down Argument that I discuss below will also arise from McKenna’s alternative. This is because McKenna takes free will to have “a significant role to play in grounding [an agent’s] being blameworthy and an apt target of blame” (McKenna 2019b, 253). However, the Top-Down Argument has problems making sense of the idea that free will plays this role.

3 In characterizing MP, I assume, for the sake of the argument, that List is correct when he argues that free will consists in causally efficient intentional agency and the ability to do otherwise. Moreover, I assume that blameworthiness presupposes wrongness and having sufficient reason to believe that the action is wrong. All these assumptions are controversial (see, e.g., Rosen 2004; Scanlon 2008, chap. 4). However, their truth is not crucial for the main point. If blameworthiness presupposes something different, one can easily reformulate the characterization of MP accordingly.

4 Recall that List (2019, 85) seems to reject conditional and dispositional interpretations of “can do otherwise” partly because these senses seem to be irrelevant for free will.

5 One may ask if the factors I discuss in the main text undermine the voters’ ability to do otherwise (as I suggest) or rather show that their voting behavior does not have the right kind of source for grounding blameworthiness (thanks to an anonymous referee for pressing me on this). In this paper, I cannot go into the debate about whether free will should be understood in terms of sourcehood, can do otherwise, both, or something else (for an overview, see O’Connor and Franklin 2021, sec. 2.3). Note, however, that some of the factors discussed can be understood as undermining the ability to do otherwise in some sense of “can do otherwise”. Take, for example, a reason-responsiveness account of the ability to do otherwise, according to which we can do otherwise in the relevant sense just in case there is a range of nearby and similar possible worlds in which we correctly realize and respond to the relevant normative reasons (see, e.g., Brink 2021, sec. 32). Now, let us go back to the voting case. We can imagine voters for whom it would be false to say that they are reason-responsive in this sense. Perhaps, the propaganda or manipulation was so effective that the possible worlds in which they correctly realize and respond to their moral reasons to vote for another party are far away. Then, they cannot do otherwise on this rough reason-responsiveness account of “can do otherwise”. Thus, the factors discussed above can be understood as undermining the ability to do otherwise in some sense of “ability to do otherwise”.

6 This paper profited a lot from discussions at the online workshop “Compatibilist Libertarianism: Advantages and Challenges” in October 2021 organized by Alexander Gebharter and Maria Sekatskaya. I’m grateful to the organizers and participants, especially Christian List, for helpful comments and suggestions. I’m also grateful to two anonymous reviewers for this journal who helped to make the paper better. Thanks to Claire Davis for proof reading. Work on this paper was supported by the Austrian Science Fund (FWF): P 34,851-G and is part of the research project *The Sense of Responsibility Worth Worrying About.*
